# Nitrogen Removal in a Full-Scale Domestic Wastewater Treatment Plant with Activated Sludge and Trickling Filter

**DOI:** 10.1155/2013/504705

**Published:** 2013-04-28

**Authors:** Davood Nourmohammadi, Mir-Bager Esmaeeli, Hossein Akbarian, Mohammad Ghasemian

**Affiliations:** ^1^Tehran Sewerage Company, Tehran 15688, Iran; ^2^Environment Research Center, School of Health, Isfahan University of Medical Sciences, Isfahan 8613846191, Iran

## Abstract

During the last decade, more stringent effluent requirements concerning the nutrients effluent values have been imposed by legislation and social concern. In this study, efficiency of total nitrogen removal in activated sludge and trickling filter processes (AS/TF) was investigated in Tehran North wastewater treatment plant. Biological system in this site was included, anoxic selector tank, aeration tank, final sedimentation, and trickling filter. A part of treated wastewater before chlorination was mixed with supernatant of dewatered sludge and fed to the trickling filter. Supernatant of dewatered sludge with high concentration of NH_4_-N was diluted by treated wastewater to provide complete nitrification in trickling filter Produced nitrate in trickling filter was arrived to the anoxic tank and converted to nitrogen gas by denitrification. According to the study result, low concentration of organic carbone and high concentration of NH_4_-N led to nitrification in TF, then nitrate denitrification to nitrogen gas occurred in selector area. NH_4_-N concentration decreased from 26.8 mg/L to 0.29 mg/L in TF, and NO_3_-N concentration increased from 8.8 mg/L to 27 mg/L in TF. Consequently, the total nitrogen decreased approximately to 50% in biological process. This efficiency has been observed in returned flow around 24% from final sedimentation into TF. It was concluded that, in comparison with biological nutrient removal processes, this process is very efficient and simple.

## 1. Introduction

Many industrial effluents, agricultural wastes, or other human activities generate high strength ammonium wastewater [1]. Water pollution represents one of the most serious environmental problems due to the discharge of nutrients into receiving waters. Hence, stricter standards for the operation of wastewater treatment plants (WWTPs) have been imposed by authorities. In order to meet these standards, improved control of WWTPs is needed. WWTPs should be controlled in such a way that plant operating costs are minimized, while effluent standards are still maintained. Different control algorithms for WWTPs have been introduced over the years [2]. Approximately 25% of all water body impairments are due to nutrient-related causes (e.g., nutrients, oxygen depletion, algal growth, ammonia, harmful algal blooms, biological integrity, and turbidity). Total effluent nitrogen comprises ammonia, nitrate, particulate organic nitrogen, and soluble organic nitrogen. The biological processes that primarily remove nitrogen are nitrification and denitrification [3]. Advances in secondary wastewater treatment processes and increased implementation of regulations on discharged effluents over the last few decades have led to variations in wastewater treatment ranging from simple organic carbon oxidation to the removal of nitrogen (N) and phosphorus (P) [4]. Nitrogen removal is normally realized by sequentially alternating between oxic and anoxic conditions or by the creation of separated zones with suitable conditions for nitrification and denitrification, respectively. Alternatively, high rates of simultaneous nitrification and denitrification (SND) can be achieved, in activated sludge and biofilm type systems alike, at operational conditions where both oxic and anoxic microenvironments are present. Nitrification can occur at the liquid/biomass interface, while denitrification of nitrate (or nitrite) may be found in deeper subsurface biomass zones [5].

Organic matter and nutrient removal from wastewater improves the environmental conditions as well as provides health and well-being to the citizens [6]. Activated sludge process is one of the most widely used biological treatments of wastewaters containing carbon and nitrogen pollutants [7]. Conventional nitrogen removal from wastewater by activated sludge is most often achieved by sequential aerobic and anoxic processes. There is a tremendous need to develop reliable technologies for the treatment of domestic wastewater in developing countries. Such treatment systems must fulfill many requirements, such as simple design, use of nonsophisticated equipment, high treatment efficiency, and low operating and capital costs. In addition, consonant with population growth and increase in urbanization, the cost and availability of land are becoming a limiting factor, and “footprint size” is increasingly becoming important in the choice of a treatment system [8]. There are a number of BNR process configurations available. Some BNR systems are designed to remove only TN or TP, while others remove both. For BNR systems to result in low TN and TP effluent concentrations, proper operation and control of the systems are essential. Operators should be trained to understand how temperature, dissolved oxygen (DO) levels, pH, filamentous growth, and recycle loads affect system performance. BNR costs differ for new plants and retrofits. New plant BNR costs are based on estimated influent quality, target effluent quality, and available funding [9]. One of the key design parameters for any biological nutrient removal system is the influent C, N, and P ratios [1]. The COD/N ratio influences the population of microorganisms. Since an increase in this parameter results in a decrease in nitrification efficiency and an improvement of denitrification efficiency [10]. The control of DO concentration is also crucial as it determines the dominant processes in the reactor. A high DO inhibits denitrification, whereas a low DO causes a limitation of ammonia oxidation [11]. DO is easily controlled by manipulating the air supply, and it strongly influences the cost of wastewater treatment resulting from aeration. A low COD/N ratio and a low DO promote partial nitrification, and this diminishes the oxygen demand by 25% and the COD requirement for denitrification by 40% [12]. The most important factors affecting SND are organic carbon, DO concentration, and floc size [13]. Simultaneous growth of nitrifiers (autotrophs) and heterotrophs in a single reactor with a high COD/N ratio causes low nitrification efficiency due to competition between these two bacterial groups [14]. The optimal DO concentration for effective nitrogen removal via SND equals 0.5 mg O_2_/L [15]. The aim of this study is survey of efficiency of total nitrogen removal in activated sludge and trickling filter processes (AS/TF) in Tehran North wastewater treatment plant.

## 2. Materials and Methods

### 2.1. Description of TWWTP

Tehran wastewater treatment plant (TWWTP) will consist of 4 modules, designed for 2,100,000 person and an average daily inflow of 5.2 m³/s. This plant is located within the latitude and longitude of 35.569098 and 51.439877. The two wastewater streams are combined down flow of separate flow measurements and treated together by mechanical and biological treatment steps supplemented with sludge treatment units.

Aeration tanks are employed for biological removal of pollutants using the activated sludge process. Each of the four aeration tanks consists of a selector cascade and two additional cascades. The basins are designed as plug flow reactors of rectangular shape. The outlet of the trickling filters will also be transferred into the inlet channels of the aeration tanks. During normal operation, each of the four trickling filters, will discharge its outlet in one inlet channel of the aeration tanks. Thus, equal distribution of nitrate to the four lines is provided. However, in case of maintenance work in either an aeration tank or a trickling filter it will also be possible to combine the trickling filter outlets in a combined collection/distribution structure and to distribute the flow to all lines (which are in operation) by gravity flow. The aeration tanks represent the very location in which the biological treatment of wastewater takes place by adopting the medium-loaded activated sludge process. Due to the treatment target and the system chosen, mainly degradation of BOD_5_ (organic pollutants) will be performed. The required denitrification will mainly be achieved in the anoxic selector cascade and by simultaneous denitrification in anoxic zones of the aerated part of the tank and within the sludge flocks. During periods with low wastewater temperature the predenitrification and simultaneous denitrification will provide a nitrate-free effluent. During periods with high wastewater temperature, some nitrate might remain in the effluent, which is not affecting the effluent quality as long as severe denitrification in the final sedimentation tanks is prevented. To prevent this “wild” denitrification, it will be possible to reduce the sludge retention time in the final sedimentation tanks to below 1 hour by control of the return sludge flow. A selector is employed to prevent and to control filamentous bacteria growth. Air or mechanical mixers can be used for sufficient mixing within the selector compartment. In this case, the selector will mainly provide anoxic conditions due to the inlet of nitrate from trickling filter effluent. Some air supply is foreseen for mixing purposes only. With regard to denitrification capacity, the introduced air is considered in the design as shown below. Generally, the air supply will only have a limited effect on denitrification due to the high respiration rate in this initial compartment of the aeration tank (up to 4 times higher than average respiration rate). Effluent transfers the treated water to agricultural network, in case of noncompliance of the treated water quality with the stipulated treatment standard.

### 2.2. WWTP Measurements

#### 2.2.1. Full-Scale Experiments

**Figure 1:**
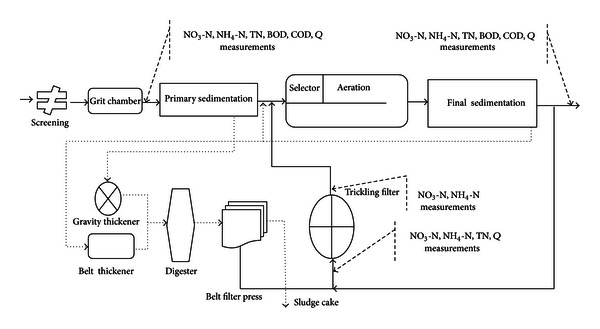
The TWWTP schematic and sampling points.

A year monitoring campaign was set up in one of the beds (March, 2011, and March, 2012), including the measurement of flow rate (in flow and out flow of the plant, tricking filter inflow) and the collection of weekly samples (one single sample by week, during 57 weeks) in four points: influent and effluent and two samples in inlet and outlet of trickling filter to determine the total nitrogen (TN), ammonia nitrogen (NH_4_-N), and nitrate nitrogen (NO_3_-N). For determination of COD/N in this study, COD sample in influent was taken.  Figure 1 shows the WWTP processes schematic and location of sampling.

The COD was determined with cuvette tests LCK 314 (15–150 mg COD/L) and LCK 514 (100–2,000 mg COD/L), following DIN 38049-4, and a CADAS 50 spectrometer (Hach Lange, Germany). Total nitrogen, ammonia nitrogen, and nitrate nitrogen were obtained using the cuvette tests LCK 238 (5–40 mg N/L), LCK 303 (2–47 mg NH_4_-N/L), LCK 342 (0.6–6 mg NO_2_-N L21), and LCK 339(0.23–13.50 mg NO_3_-N/L), following standards DIN38406-E 5-1 and DIN 38402-A51 and the same spectrometer. BOD was determined according to the standard methods for the examination of water and wastewater [16]. The experimental apparatus consisted of three parallel module with electrodes for a continuous monitoring of pH, temperature and dissolved oxygen (DO), and computer control system to maintain DO concentration and temperature around set points.

## 3. Result and Discussion

**Figure 2:**
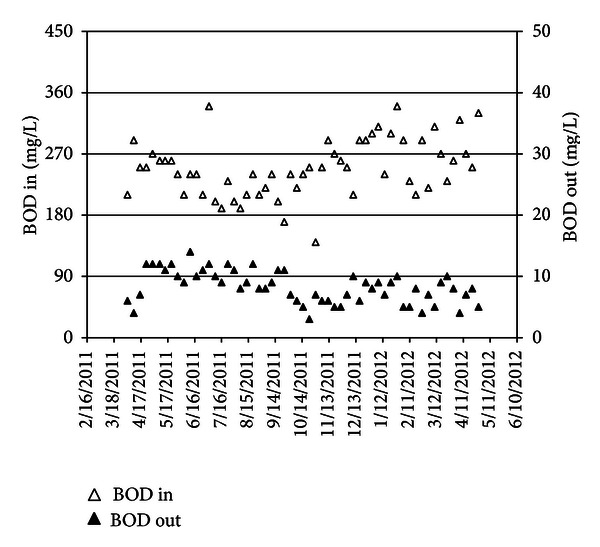
BOD_5_ in influent and effluent.

**Figure 3:**
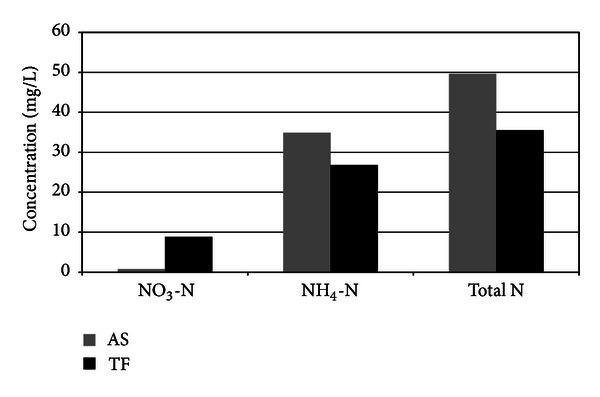
NO_3_-N, NH_4_-N, and total nitrogen in AS and TF.

Sewer system which transfers sewerage to Tehran North wastewater treatment plant is too long and reaches to more than 30 kilometers in some branch. Height deference between the highest and lowest levels in above mention system is more than 700 meters. This matter causes the BOD_5_ concentrating in sewer to decrease by reaeration. Average concentrations of BOD_5_ in influent and effluent of the plant were, respectively, 255 mg/L and 8.2 mg/L per studied duration. In such condition, ammonification also happens (see Figure 2). Between NH_4_-N, NO_3_-N, and TN in raw wastewater is shown in Figure 3.

**Figure 4:**
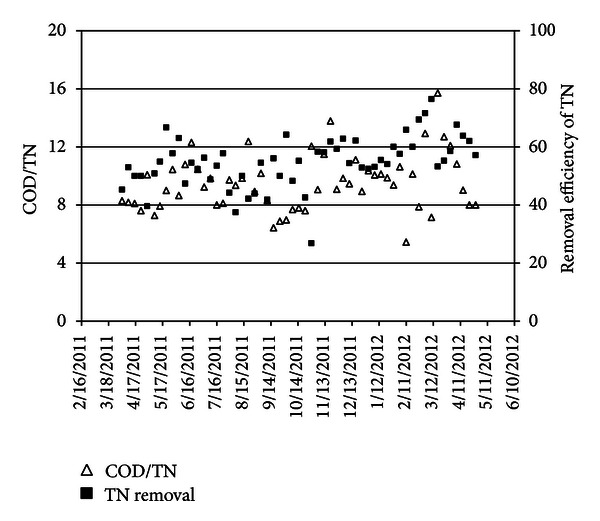
COD/TN and TN removal efficiency in influent.

Due to the importance of COD/TN, this factor and its trend was calculated. In NH4-N was analyzed in inlet and outlet of trickling filter, and that removal efficiency was 98%. Between TN removal efficiency and COD/TN shows that these parameters are not related directly, and this trend implies the effective parameters on the total nitrogen removal efficiency. The proof which approves the above result is the stable removal of BOD_5_ and unstable removal efficiency of total nitrogen as shown in Figure 4.

**Figure 5:**
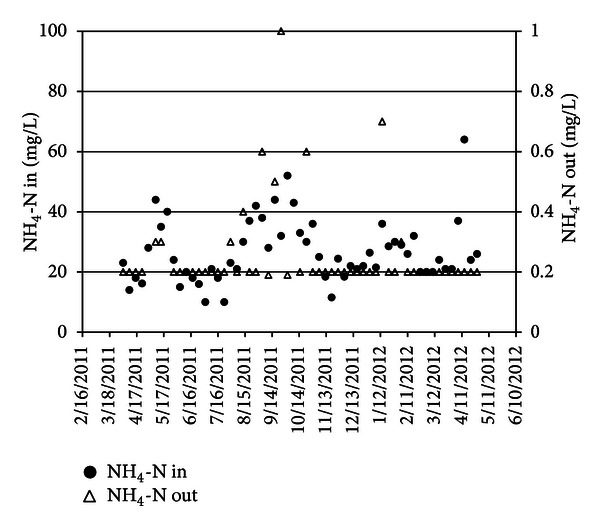
NH_4_-N in before and after TF.

Concentration of NH_4_-N before trickling filter and after aeration tank has shown that nitrification was well done. NH_4_-N concentration decreased from 26.8 mg/L to 0.29 mg/L in TF (see Figure 5), and NO_3_-N concentration increased from 8.8 mg/L to 27 mg/L in TF.

Since, the main inflow of trickling filter is supplied by effluent, absorbed oxygen in this unit is used for converting NH_4_-N to NO_3_-N, and there is no requirement to consume oxygen for organic carbon oxidation. Base on Figure 7, average ratio for TF inflow to total plant flow was 24% and data approves that this ratio is desirable for 50% of TN removal.

**Figure 6:**
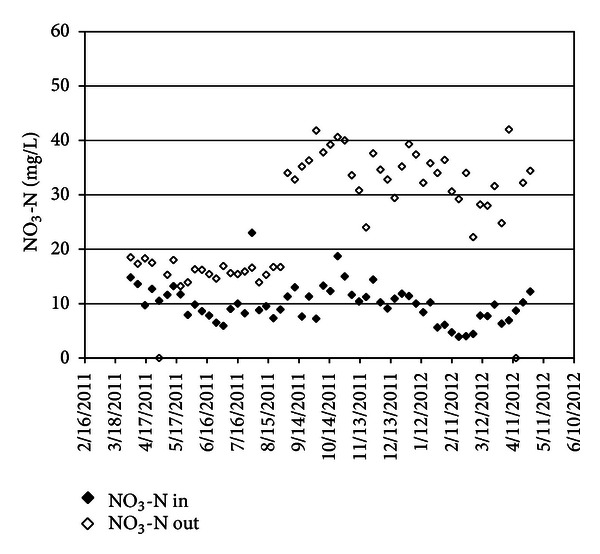
NO_3_-N in before and after TF.

Average concentration of total nitrogen of Tehran wastewater treatment plant was analyzed and was shown to be 55 mg/L of which 35 mg/L is related to NH_4_-N and 0.8 mg/L for NO_3_-N as in Figure 6.

**Figure 7:**
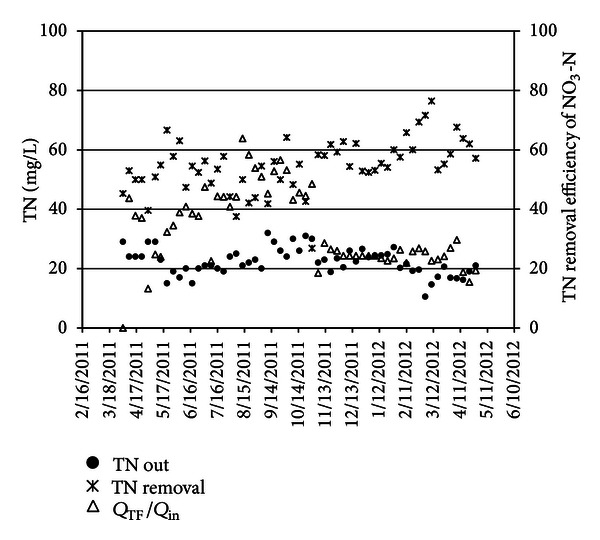
Removal efficiency of TN, *Q*_TF_/*Q*_in,_ and TN out.

**Figure 8:**
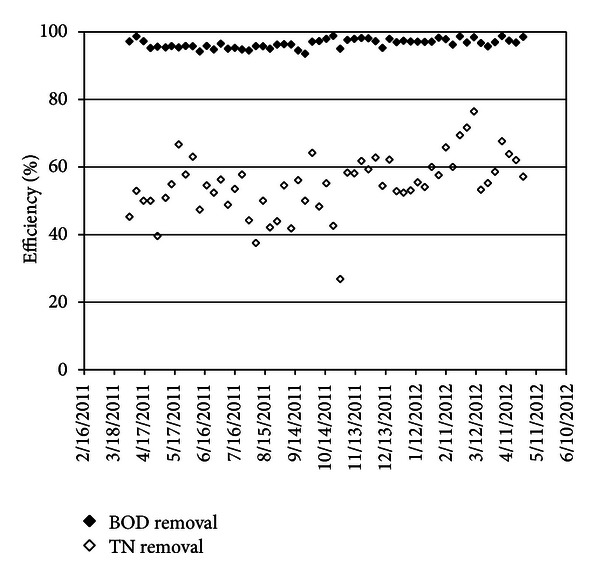
BOD_5_ and TN removal efficiency.

Relation between tricking filter flow to total plant flow ratio and total nitrogen removal efficiency has been demonstrated in Figure 7, in which the average of *Q*_TF_/*Q*_in_ reaches to 24%. This figure shows that nitrification and denitrification have been done well, and dissolve oxygen has been available and enough for nitrification in trickling filter. In order to evaluate TN removal efficiency, this parameter is measured in before and after biological units Based on the plant design, at least 50% of TN must be removed. Therefore, trickling filter was used, and the same time, the power consumption also decreased. Reduction of energy consumption was related to lower dissolve oxygen in aeration tanks; the concentration of dissolve oxygen in aeration tank is set to 0.9 mg/L, and under this condition, BOD_5_ and TN diminished to the defined limit. Also trickling filters are employed for the nitrification of ammonia contained in the highly concentrated filtrate from sludge dewatering Figure 8 illustrates BOD and TN removal efficiency. Additionally, a part of the effluent of the activated sludge stage is returned via the trickling filters for nitrification. Due to subsequent denitrification of the produced nitrate in the activated sludge stage, the effluent requirement of N_total_ ≤ 30 mg/L will be achieved. Beside the transfer of ammonia to the trickling filters, the recirculation of final clarifier effluent also serves for dilution of the sludge dewatering filtrate in order to provide sufficient alkalinity in the trickling filters. Differing from the original design, it is now foreseen to transfer only filtrate from sludge dewatering to the trickling filters. All other sludge waters and wash waters will be transferred to the primary sedimentation or to the activated sludge stage. Due to this process adaptation, the BOD_5_ load and suspended solids (SSs) load to the trickling filters will be very low.

Albuquerque et al. reported that ammonia removal rates reached higher values (from 172 g N/m^3^ d to 564 g N/m^3^ d) for C/N = 2 and lower values (from 13.6 g N/m^3^ d to 34.6 g N/m^3^ d) for C/N = 20. Between 50% and 70% of the ammonia was removed in the upper section of the filter [17].

In Ryu et al. study, NH_4_-N removal performance was stable with the removal efficiency of 95-96% on average, regardless of HRT applied. At the TCOD- to TKN-ratio of 3 : 6 of four-stage biological aerated filter system, the effluent NO_3_-N concentration of denitrification column was 2.7 mg/L which was excellent in nitrogen removal performance by employing four columns functioning as sorption, nitrification, denitrification, and purification, respectively [18].

Based on Timothy et al. study, in various operating condition, the TFs removed an average of 2.4 × 10^−4^ kg N/(dm^2^), and the NTFs removed an average of 1.5 × 10^−5^ kg N/(dm^2^) because of low loading [19].

In the full-scale bioreactor for treating an average leachate flow of 304 m^3^ d^−1^ with a SRT range of 12 and 18 d, the average COD, NH_4_^+^-N, and NO_3_^−^-N concentrations at the upstream end of the bioreactor, that is, influent, were 554, 634, and 3 mg L^−1^, respectively, whereas no NO_2_^−^-N was detected in the influent [20].

The resulting independence of the trickling filter from possible fluctuations of the quality of other process streams is a further advantage. Due to the low BOD_5_ and SS load, it is evident that the main target of this treatment step is the oxidation of ammonia only. Some degradation of organic pollutants can be seen as side-effect, and it should be stressed that with the low organic load figures TF clogging is definitely prevented. The biologically active biomass will adhere to the filter material and will create a biological lawn mainly consisting of nitrifying bacteria (*Nitrosomonas* and *Nitrobacter*), which will accomplish the oxidation of ammonia to nitrate and further to nitrate. After passing the filter material, the nitrified water is collected and transferred to the inlet of the aeration tank by gravity. 

During normal operation, each trickling filter will be directly connected with the inlet channel of one aeration tank. For redundancy reasons, it will also be possible to combine all trickling filter outlet flows before distribution to the aeration tanks. The recycle of the trickling filter effluent to the activated sludge stage contributes to an overall low energy demand of the biological treatment stage.

## 4. Conclusion

In AS/TF system, low concentration of organic carbone and high concentration of NH_4_-N led to nitrification in TF part, then nitrate denitrification to nitrogen gas occurred in selector area. So, these advantages in TF part causes a better performance than for TN removal in TF and AS separately. NH_4_-N concentration decreased from 26.8 mg/L to 0.29 mg/L in TF, and NO_3_-N concentration increased from 8.8 mg/L to 27 mg/L in TF. Consequently, total nitrogen decreased approximately to 50% in biological process. This efficiency has been observed in returned flow around 24% from final sedimentation into TF. It was concluded that, in comparison with biological nutrient removal processes, this process is very efficient and simple.
